# Utilizing Repetitive Serial Placental Ultrasounds for Diagnosing a Massive Subchorionic Thrombohematoma: Insights Into Soluble FMS-Like Tyrosine Kinase-1/Placental Growth Factor (sFlt-1/PlGF) Trends

**DOI:** 10.7759/cureus.63927

**Published:** 2024-07-05

**Authors:** Takeshi Nagao, Akihiro Hasegawa, Osamu Samura, Aikou Okamoto

**Affiliations:** 1 Department of Obstetrics and Gynecology, The Jikei University School of Medicine, Tokyo, JPN

**Keywords:** pregnancy, neonatal death, hypertensive disorder of pregnancy, fetal growth restriction, massive subchorionic thrombohematoma

## Abstract

The patient, a 34-year-old primigravida with no prior medical history, presented at 23 + 0 weeks with gestational hypertension and fetal growth restriction (FGR). Ultrasound examination showed a placental mass, and subsequent repeated ultrasound scans revealed changes in the mass' echogenicity, raising suspicion of a massive subchorionic thrombohematoma (MST). While the blood pressure was mildly elevated without proteinuria and organ dysfunctions, serum soluble fms-like tyrosine kinase-1/placental growth factor (sFlt-1/PlGF) ratios showed significantly elevated values. A cesarean section was performed at 29 + 2 weeks due to the nonreassuring fetal status. The female infant, with Apgar scores of 1/1 at one/five minutes and an umbilical artery pH of 7.16, remained unresponsive and died seven hours postdelivery. Pathology examination revealed a massive hematoma in the subchorionic space, measuring 22 mm thick, directly beneath the umbilical cord attachment. This case underscores the importance of repetitive placental ultrasound in MST diagnosis and suggests the potential utility of sFlt-1/PlGF ratios in predicting adverse outcomes.

## Introduction

Massive subchorionic thrombohematoma (MST) is rare, with its precise frequency remaining unclear. Initially reported as Breus’ mole in 1892, MST is characterized by a large maternal blood clot in the subchorionic space that extends into and dissects the overlying chorionic plate [1]. In most patients with MST, diagnosis is prompted by the presence of a placental mass, with a thickness exceeding 10 mm commonly used as a criterion [2]. The condition is associated with an increased risk of adverse perinatal outcomes such as preterm birth, fetal growth restriction (FGR), intrauterine fetal demise, and neonatal death [2-3]. However, reports on MST are limited to a few case reports or case series, and poor prognostic factors in MST patients, the appropriate diagnostic methods, determination of delivery timing, and perinatal management for MST are not yet fully understood [4-7]. We present a case initially managed as a hypertensive disorder of pregnancy (HDP) with FGR, which was subsequently diagnosed as MST through repeated detailed placental ultrasounds. Additionally, we report, for the first time, the trend of maternal serum soluble fms-like tyrosine kinase-1/placental growth factor (sFlt-1/PlGF) ratio during pregnancy in MST patients.

## Case presentation

**Figure 1:**
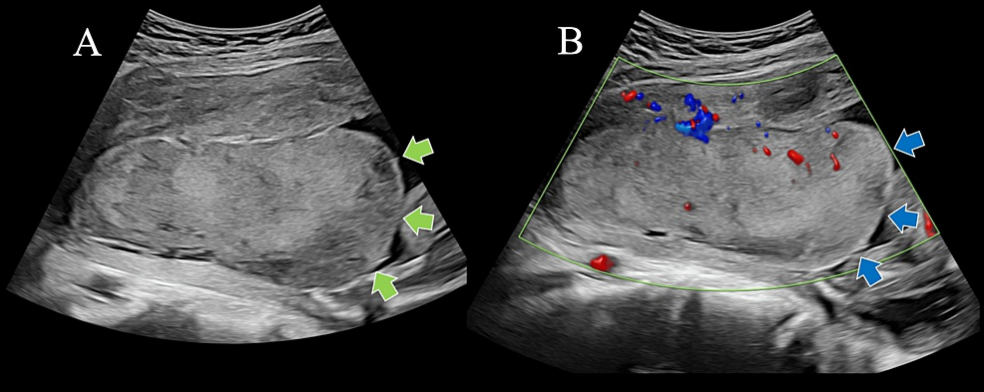
Changes in echogenicity of the placental mass (A) A placental mass (green arrow) with confirmed low echo density on ultrasound at 23 + 0 weeks of gestation. (B) A placental mass (blue arrow) with changed echogenicity at 23 + 2 weeks of gestation.

The patient, a 34-year-old primigravida with no medical history, had conceived through artificial insemination by her husband. She was referred to our hospital at 23 + 0 weeks of gestation due to HDP with FGR. Ultrasonography revealed an estimated fetal weight of 318 g (0.1 percentile) with no additional abnormalities. The maternal blood tests for toxoplasmosis, rubella cytomegalovirus, herpes simplex, and HIV (TORCH) infections were all negative. Her blood pressure was elevated (149/95 mmHg), with no indications of proteinuria, thrombocytopenia, or elevated liver enzymes. A placental ultrasound indicated a placental mass with low echogenicity, which had not been noted at the previous hospital (Figure 1A). Thus, placental tumors were considered. However, two days later, the echogenicity of the mass was high, and on another day, it changed to mixed high and low. A suspicion of MST arose from the changes in echogenicity of the placental mass (Figure 1B). Laboratory tests at 23 + 6 weeks showed an elevated sFlt-1 (8020 pg/mL) and a reduced PlGF (13.4 pg/mL), with a ratio of 599. 

**Figure 2:**
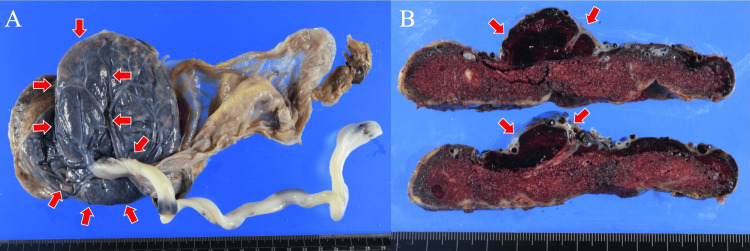
Massive hematoma (A)-(B) A massive hematoma (red arrow) measuring 105 mm × 55 mm in the subchorionic space, with a thickness of 22 mm, located directly beneath the umbilical cord attachment

**Figure 3:**
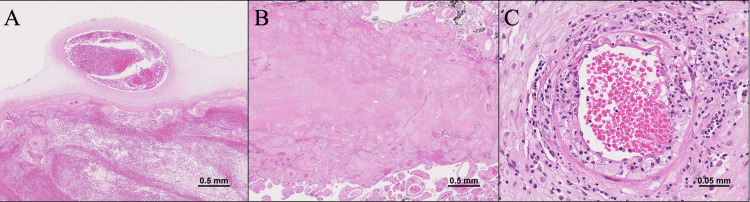
Pathological findings of the placenta (A)-(B) Pathological examination of the placenta revealed a hematoma beneath the chorionic plate, surrounded by numerous microinfarcts. (C) Decidual vasculopathy was also identified

Counseling was provided to the patient and her husband regarding the possibility of MST and its associated poor perinatal outcomes. Subsequently, the echogenicity of the placental mass gradually ceased to change, becoming either the same as or lower in echo than that of the placenta. At 28 + 6 weeks, laboratory tests showed further deterioration in sFlt-1 (11340 pg/mL) and PlGF (5.1 pg/mL), with a ratio of 2610. However, the rise in maternal blood pressure was mild (142/98 mmHg) without antihypertensive medication. Additionally, there was no proteinuria, thrombocytopenia, or elevated liver enzymes. The maximum vertical pocket was maintained at 21 mm. Daily nonstress test monitoring was conducted, and the evaluation of biophysical profile scoring and fetal doppler of the umbilical artery, middle cerebral artery, and ductus venosus was performed 2-3 times a week. Due to a ductus venosus a-wave at baseline and reverse end-diastolic flow in the umbilical artery, the patient underwent a cesarean section at 29 + 2 weeks after receiving corticosteroids for fetal lung maturation and magnesium sulfate for fetal neuroprotection. A female infant weighing 515 g (<3rd percentile) was delivered without malformations. Apgar scores at one and five minutes were 1 and 1, respectively, and umbilical arterial blood pH was 7.16. Despite resuscitative efforts, the neonate remained unresponsive and died seven hours postdelivery. The placenta measured 11.0 × 10.5 × 2.6 cm and weighed 168 g(<10th percentile). The umbilical cord had a diameter of 0.8 cm and a length of 26 cm. Placental examination revealed a massive hematoma (105 mm × 55 mm) in the subchorionic space, measuring 22 mm in thickness, located directly beneath the umbilical cord attachment (Figure 2A-2B). In the pathological examination of the placenta, a hematoma was observed beneath the chorionic plate, surrounded by numerous microinfarcts (Figure 3B-3C). Decidual vasculopathy was also identified (Figure 3A).

Maternal blood pressure increased further postdelivery and was managed with nifedipine 40 mg/day for two weeks. There were no abnormalities in her hepatic or renal function, thrombocytopenia, or proteinuria. Three months postpartum, thrombogenicity and coagulability tests were performed to investigate any underlying maternal conditions that could cause coagulation abnormalities or a thrombotic tendency. All results were normal, and conditions such as thrombophilia and antiphospholipid syndrome were not suspected.

The patient conceived again one year later and was prescribed 100 mg/day of low-dose aspirin from 10 weeks to 36 weeks of gestation. She delivered a healthy male infant weighing 2625 g (19.4 percentile) at 38 weeks and zero days. There were no abnormal findings in the placenta, and MST did not recur. No other perinatal complications were observed.

## Discussion

This case initially presented as FGR accompanied by mild hypertension. However, the identification of a placental mass on ultrasound, alongside changes in echogenicity upon repeated observations, led to the diagnosis of MST. Furthermore, this case marks the first report detailing the trend in the sFlt-1/PlGF ratio in an MST patient. Clinically, this case underscores two crucial educational points. Firstly, it emphasizes the importance of not overlooking MST despite its poor prognosis, highlighting the necessity for detailed and repeated placental ultrasound examinations for diagnosis. Secondly, it highlights the potential utility of the sFlt-1/PlGF ratio in predicting the prognosis of MST patients. 

The poor fetal/neonatal prognosis associated with MST underscores the imperative of not overlooking this condition. Therefore, detailed placental ultrasound, conducted repeatedly, is crucial. In the largest case series of MST, out of 14 cases, 13 resulted in preterm delivery, with viable infants obtained in only half of these cases [3]. Similarly, in another case series involving 10 cases, eight deliveries occurred before 37 weeks, with only one resulting in a viable infant without small for gestational age (SGA) or FGR [2]. Various echogenic findings have been reported in these MST placental masses, suggesting hematomas, such as a homogeneous mass, heteroechoic collection, hypodense area, and hypoechoic cystic area in the placenta [2]. The diversity of reported findings in these masses likely stems from the fact that hematomas undergo cycles of microbleeding and clotting, leading to changes in echogenicity depending on the timing of the examination. Consequently, the echogenicity of surrounding placental tissue and the hematoma may become similar, making it challenging to distinguish between them. This highlights the importance of vigilant and repeated assessments of placental masses. 

Risk factors for poor prognosis in MST patients have not been firmly established; however, the sFlt-1/PlGF ratio may provide valuable insight. This case marks the first instance in which the sFlt-1/PlGF ratio was monitored in an MST patient. A high sFlt-1/PlGF ratio indicates placental insufficiency due to hypoxia/ischemia and serves as a predictor for the onset of preeclampsia and adverse fetal/neonatal outcomes [8-10]. In this case, the timing of delivery was determined based on observations of Doppler velocimetry in the ductus venous and umbilical artery. Despite reports suggesting the potential necessity of imminent delivery for sFlt-1/PlGF values ≥ 655, in this particular case, the ratio surged to 2610 just before delivery [11]. We hypothesized two causes of severe placental insufficiency in this case. Firstly, the presence of a hematoma directly beneath the umbilical cord attachment may have increased intrasubchorionic pressure, compressing small vessels and potentially leading to severe hypoxia/ischemia. Secondly, the lack of change in hematoma size during pregnancy could have contributed to hypoxia/ischemia. In some MST cases, the size of the hematoma (mass) may vary during pregnancy due to bleeding and absorption [12]. Examination of the placenta after delivery, in this case, revealed the hematoma to be extremely firm, suggesting it remained unabsorbed during pregnancy, potentially exacerbating hypoxia/ischemia. 

This instance underscores the significance of detecting undiagnosed MST through repeated, detailed placental ultrasound evaluations. Additionally, there is potential for further exploration of the utility of the sFlt-1/PlGF ratio in predicting adverse outcomes for MST patients. With the accumulation of more reports like this one, we can anticipate the emergence of high-quality evidence concerning the etiology and pathophysiology of MST, as well as appropriate management strategies and the optimal timing of delivery.

## Conclusions

The diagnosis and management of MST present significant challenges due to the severe risks it poses to fetal and neonatal health. This case highlights the critical role of repeated detailed placental ultrasounds in identifying MST, given the changing echogenicity of the placental mass over time. Moreover, the elevated sFlt-1/PlGF ratios observed in this case may suggest their potential utility as biomarkers for predicting adverse outcomes in MST patients. Further research is warranted to establish robust diagnostic criteria and management strategies for MST to improve perinatal outcomes.
